# A comparative evaluation of PCR- based methods for species- specific determination of African animal trypanosomes in Ugandan cattle

**DOI:** 10.1186/1756-3305-6-316

**Published:** 2013-11-01

**Authors:** Heba A Ahmed, Kim Picozzi, Susan C Welburn, Ewan T MacLeod

**Affiliations:** 1 Division of Pathway Medicine and Centre for Infectious Diseases, School of Biomedical Sciences, College of Medicine and Veterinary Medicine, The University of Edinburgh, Chancellor’s Building, 49 Little France Crescent, Edinburgh, EH16 4SB, UK; 2 Faculty of Veterinary Medicine, Zagazig University, Zagazig 44611, Egypt

**Keywords:** Trypanosome, Brucei, Congolense, Vivax, Species-specific PCR, ITS PCR, Nagana

## Abstract

**Background:**

In recent years, PCR has been become widely applied for the detection of trypanosomes overcoming many of the constraints of parasitological and serological techniques, being highly sensitive and specific for trypanosome detection. Individual species-specific multi-copy trypanosome DNA sequences can be targeted to identify parasites. Highly conserved ribosomal RNA (rRNA) genes are also useful for comparisons between closely related species. The internal transcribed spacer regions (ITS) in particular are relatively small, show variability among related species and are flanked by highly conserved segments to which PCR primers can be designed. Individual variations in inter-species length makes the ITS region a useful marker for identification of multiple trypanosome species within a sample.

**Methods:**

Six hundred blood samples from cattle collected in Uganda on FTA cards were screened using individual species-specific primers for *Trypanosoma congolense*, *Trypanosoma brucei* and *Trypanosoma vivax* and compared to a modified (using eluate extracted using chelex) ITS-PCR reaction.

**Results:**

The comparative analysis showed that the species-specific primer sets showed poor agreement with the ITS primer set. Using species-specific PCR for *Trypanozoon,* a prevalence of 10.5% was observed as compared to 0.2% using ITS PCR (Kappa = 0.03). For *Trypanosoma congolense*, the species-specific PCR reaction indicated a prevalence of 0% compared to 2.2% using ITS PCR (Kappa = 0). For *T. vivax*, species-specific PCR detected prevalence of 5.7% compared to 2.8% for ITS PCR (Kappa = 0.29).

**Conclusions:**

When selecting PCR based tools to apply to epidemiological surveys for generation of prevalence data for animal trypanosomiasis, it is recommended that species-specific primers are used, being the most sensitive diagnostic tool for screening samples to identify members of *Trypanozoon (T. b. brucei* s.l). While ITS primers are useful for studying the prevalence of trypanosomes causing nagana (in this study the species-specific primers did not detect the presence of *T. congolense*) there were discrepancies between both the species-specific primers and ITS for the detection of *T. vivax*.

## Background

Routine diagnosis of trypanosomiasis using classical parasitological approaches shows poor sensitivity under field conditions [1]. This is in part, due to the normally very low peripheral parasitemia of naturally infected animals [2,3]. The limitations of microscopy for both human and animal trypanosomiasis screening led to the development of a range of serological tests: such as the complement fixation test (CFT), indirect fluorescent antibody test (IFAT), card agglutination test (CATT) and enzyme linked immunosorbant assay (ELISA). However, these methods are unable to differentiate between existing infections and previous exposure to infection, and may lack specificity [1,4].

In recent years, PCR has been widely applied for the detection of trypanosomes and has been shown to be highly sensitive and specific in the laboratory [5]. The use of PCR for detecting trypanosome DNA is the most reliable and accurate technique available for the specific identification of natural animal infections for most trypanosome species and sub-species [6,7]. Species-specific DNA targets have been identified for the most important pathogenic trypanosome species that occur in cattle (*Trypanosoma brucei* s.l., *Trypanosoma congolense* and *Trypanosoma vivax*) and PCR based methods for their amplification developed.

For detection of *Trypanozoon* (*Trypanosoma brucei brucei, Trypanosoma brucei gambiense, Trypanosoma brucei rhodesiense, Trypanosoma evansi* and *Trypanosoma equiperdum*), the most common target is the 177 bp DNA satellite repeat sequence originally described by Sloof *et al*. [8] that exists in high copy number in the parasite genome (10,000). The TBR universal primers developed to amplify the 177 bp sequences from *Trypanozoon* genomic DNA, were able to detect 0.1 pg of parasite DNA, *i.e.* the amount of DNA equated to that present in a single trypanosome [9]. Weaker bands containing the 177 bp amplicon, could be detected when the DNA content was as low as 0.01 pg (equivalent to 1/10th that of a single parasite). Clausen *et al*. [10] found the TBR primer set able to detect as little as one femtogram of purified DNA (which is equivalent to about 1% of the genome content of one parasite). In practice, in most cases the universal primers for *Trypanozoon* are used to identify *T. b. brucei* as a first stage in the process of identifying human infective parasites within an animal population. To further discriminate *T. b. brucei* from *T. b. rhodesiense* PCR reactions targeting the single copy serum-resistance-associated (*SRA*) gene [11] have been developed and applied [6]. To permit discrimination between samples carrying human serum resistant parasites and non human infective parasites a multiplex PCR that simultaneously detects the *SRA* gene as well as the gene for phospholipase C (*GPI-PLC*) was developed [12]. Both of these single copy genes are present in *T. b. rhodesiense*, but only GPI-PLC is present in *T. b. brucei*, so if a sample shows positive amplification for GPI-PLC this indicates that sufficient *T. brucei* s.l. genomic material is present to detect a single copy gene while the presence or absence of SRA determines whether *T. b. rhodesiense* is present.

*Trypanosoma congolense* (savannah) is a highly pathogenic trypanosome that is the most widespread, in terms of both geographical and host range across Sub Saharan Africa [13]. Specific primers designed to detect *T. congolense,* which target a satellite sequence of 316 bp have been developed [9,14]. The sensitivity of the universal primers to detect *T. congolense* savannah was 0.1 pg of parasite DNA, again the amount of DNA in a single trypanosome and weak bands containing the amplification product could be detected when the DNA content was 0.01 pg or an estimated 1/10th that of a single parasite [9].

*Trypanosoma vivax* can be identified using universal primers targeting a fragment of the gene encoding *T. vivax* specific antigen [15,16]; this antigen was recognised by a monoclonal antibody (Tv27) in an Ag-ELISA. The cloned gene was found to comprise a tandem repeat, with a monomeric unit length of 900 bp in the genome of all *T. vivax* isolates from diverse geographic locations in Africa and South America.

These species-specific primers show good sensitivity and specificity against their target species. However, to screen large numbers of samples such as are derived from naturally infected hosts to determine prevalence data, multiple PCRs need to be undertaken for each sample which is both time consuming and expensive. It would be desirable to be able to apply a single PCR reaction that could simultaneously differentiate all of the trypanosome species present within a sample, making screening of a large number of samples viable in terms of time and cost.

Ribosomal RNA (rRNA) genes are highly conserved and have been proved useful for comparisons of closely related species. Eukaryotic rRNA genes occur as tandem repeats of units separated by a non-transcribed spacer (NTS) region and internal transcribed spacer regions (ITS); the ITS regions are relatively small, show variability among related species and are flanked by highly conserved segments to which PCR primers can be designed. Individual variations in inter-species length makes the ITS region a useful marker for species differentiation in trypanosomes and this and their high copy number, of around 200 makes the (ITS) a useful target for species differentiation in trypanosomes and other species [17,18]. Njiru *et al*. [19] and Cox *et al*. [20] have developed ITS-PCR methods that generate a uniquely sized PCR product for each species of trypanosome (the inter-species length variation of the ITS region of ribosomal genes). The protocol developed by Njiru *et al*. [19] is a single round PCR while Cox *et al*. [20] is a nested PCR designed to increase sensitivity and specificity. ITS PCR permits multiple trypanosome species to be detected from a single PCR reaction and reduces the cost substantially. Thumbi *et al*. [21] compared species-specific primer sets with the ITS reactions of Njiru *et al*. [19] and Cox *et al*. [20] and found no difference for the diagnosis of *Trypanozoon* and *T. congolense* but reported more *T. vivax* positives using the ITS primer sets than were derived using the species-specific primers. The species specific primer set had been developed for West African *T. vivax* isolates and this could explain the lower levels of positives generated and therefore the disagreement between the ITS PCR tests and species-specific PCR when applied to East African samples. In disagreement with the work of Thumbi *et al*. [21] a large study of domestic animals from Kenya [22], showed that the species-specific primers of Moser *et al*. [9] were significantly more sensitive and specific than the ITS PCR primers of Cox *et al*. [20].

Although FTA cards present an efficient method of collection, storage and transportation of field material using one punch to seed the PCR reaction can reduce the chances of a positive PCR results [23]. This is due to uneven distribution of parasite DNA on the card and that in order to increase the sensitivity of the PCR an increased number of punches from a card should be tested. However, this is both costly and time consuming. In order to improve the sensitivity of PCR from FTA cards, Ahmed *et al*. [24] have recently shown that through the use of an elution step simultaneously from 10 punches (each 0.2 mm) using Chelex 100® shows higher sensitivity compared to PCR of 10 punches separately. Therefore in the current work we modified the PCR designed by Cox *et al*. [20] to include a Chelex 100® elution stage in order to make the source material comparable.

In this study we have extensively tested a substantive number of cattle samples from Uganda to compare the application of species-specific primer sets targeting *Trypanozoon*[9,12], *T. congolense*[14] and *T. vivax*[14,15] with the Chelex 100® modified ITS-PCR reaction of Cox *et al*. [20].

## Methods

### Sample collection

A total of 600 blood samples were available for testing that had been collected from cattle in Uganda in 2006 from baseline sampling for the Stamp out Sleeping Sickness campaign [25]. Samples came from the following villages, Agwea (Apac district), Abuong B, Amuko Aola, Onyakede (Lira district) and Omid (Kabermaido district). Blood (around 200 μl) was collected from the ear vein and placed on an FTA®card and allowed to dry for 24 hours as described previously [3,24].

### Ethical approval

At the national and district levels, the study was conducted with the approval of the Coordinating Office for Control of Trypanosomiasis in Uganda (COCTU) as well as the District Veterinary Officers (DVOs) in each of the study districts.

### Preparation of samples

In order to allow comparison between the species-specific and the ITS-PCR of Cox *et al*. [20] the protocol of the latter was modified by the addition of a Chelex extraction as described by Becker *et al*. [26]. Ten discs (each 0.2 mm) of the FTA matrix that contain the blood sample were cut and prepared according to the manufacturer’s instructions. In order to reduce carryover contamination five blank punches were taken after each sample punch. After washing and drying the discs, DNA was eluted by heating the discs for 30 minutes at 90°C in 60 μl of 5% (w/v) aqueous suspension of Chelex 100® resin (sodium form, 50–100 dry mesh, Sigma) [26]. For PCR, 5 μl of the eluate was added to 20 μl of the PCR master mix as described by Ahmed *et al*. [24] for both the species-specific PCRs and also the modified reaction of Cox *et al*. [20]. The second round of the nested ITS-PCR was seeded with 1 μl from the first round.

The species-specific PCR reactions used in this study (see Table 1) detect *Trypanozoon* DNA (TBR-PCR [9] and GPI-PLC-PCR [12]), *T. congolense* DNA (TCS-PCR, TCF-PCR, TCK-PCR [9,14]) and *T. vivax* DNA (TV-PCR [14,15]). The band sizes amplified by the ITS-PCR of Cox *et al*. [20] are shown in Table 1.

**Table 1:** Different PCR reactions used in the current study

**PCR (reference)**	**Diagnosed species**	**Product size (bp)**	**Primer sequence**	**Number of cycles**	**Reaction conditions**
GPI-PLC-PCR [12]	*Trypanozoon*	324 bp	657: 5’- CGC TTT GTT GAG GAG CTG CAA GCA-3’	40	95°C for 15 min then 94°C for 30 sec, 63°C for 90 sec, 72°C for 70 sec, 72°C then for 10 min.
			658: 5’- TGC CAC CGC AAA GTC GTT ATT TCG-3’		
TBR-PCR [9]	*Trypanozoon*	173 bp	TBR1: 5’-CGA ATG AAT ATT AAA CAA TGC GCA GT-3’	35	94°C for 3 min then 94°C for 1 min, 55°C for 1 min, 72°C for 30 sec, then 72°C for 5 min.
			TBR2: 5’-AGA ACC ATT TAT TAG CTT TGT TGC-3’		
TCS-PCR [14]	*T. congolense* savannah	316 bp	TCS1: 5’-CGA GAA CGG GCA CTT TGC GA-3’	35	94°C for 3 min then 94°C for 1 min, 60°C for 2 min, 74°C for 30 s.
			TCS2: 5’-GGA CAA ACA AAT CCC GGGCA CA-3’		
TCK [14]	*T. congolense* kilifi	294 bp	TCK1: 5’- GTG CCC AAA TTT GAA GTG AT-3’	35	94°C for 3 min then 94°C for 1 min, 60°C for 2 min, 74°C for 30 s.
			TCK2: 5’- ACT CAA AAT CGT GCA CCT CG-3’		
TCF [14]	*T. congolense* forest	350 bp	TCF: 5’- GGA CAC GCC AGA AGG TAC TT-3’	35	94°C for 3 min then 94°C for 1 min, 60°C for 2 min, 74°C for 30 s.
			TCF2: 5’- GTT CTC GCA CCA AAT CCA AC-3’		
TV-PCR [16]	*T. vivax*	400 bp	ILO1264: 5’-CAG CTC GGC GAA GGC CAC TTC GCT GGG GTG-3’	35	94°C for 90 s min, 55°C for 90 s, 72°C for 2 min.
			ILO1265: 5’-TCG CTA CCA CAG TCG CAA TCG TCG TCT CAA GG-3’		
West African TV-PCR [15]	*T. vivax*	175 bp	TVW1: 5’-GTG CTC CAT GTG CCA CGT TG-3’	35	94°C for 1 min, 55°C for 1 min, 72°C for 2 min.
			TVW2: 5’-CAT ATG GTC TGG GAG CGG GT-3’		
ITS-PCR [20]	*T. congolense* forest	1513 bp	ITS1: 5'-GAT TAC GTC CCT GCC ATT TG-3'	Two rounds, 35 cycles, each, the second round PCR was seeded with 1 μl from the first round	95°C for 7 minutes then 94°C for 1 min, 55°C for 1 min, 72°C for 2 minutes.
	*T. congolense* kilifi	1422 bp			
	*T. congolense* savannah	1413 bp	ITS2: 5'-TTG TTC GCT ATC GGT CTT CC-3'		
	*T. brucei* s.l.	1207-1224 bp	ITS3: 5'-GGA AGC AAA AGT CGT AAC AAG G-3'		
	*T. theileri*	988 bp	ITS4: 5'-TGT TTT CTT TTC CTC CGC TG-3'		
	*T. simiae* Tsavo	954 bp			
	*T. simiae*	850 bp			
	*T. vivax*	611 bp			

One positive control (genomic DNA) for each trypanosome species was used in the corresponding species specific PCR, while for ITS-PCR, a positive control of *T. brucei* s.l was used. Negative controls consisting of water only instead of eluate and washed blank discs were run with each PCR.

Amplification products were resolved in 1% (w/v) agarose gels along with 100 bp molecular weight Superladder (ABgene, Epsom, Surrey, UK). The agarose gel was prepared in 1 × TBE (89 mM Tris-Borate, 2 mM EDTA, and pH 8.3) (Sigma-Aldrich, Poole, Dorset, UK) stained with 5 μM ethidium bromide. The gels were run in 1xTBE, 5 μM ethidium bromide for at least 45 minutes at 100 volts and visualized under an ultra violet transilluminator (Gel-Doc 2000, Bio-Rad).

When the amplification reaction gave a signal of the expected size according to the set of primers used without any signal in the negative control, infection was considered to be confirmed.

### Data analysis

Kappa values were used to determine the level of agreement between the diagnostic tests [27,28]. A Fleiss’s Kappa from rating scores using the website of Statstodo.com (http://www.statstodo.com/CohenKappa_Pgm.php) was used to compare the three PCR reactions targeting the *Trypanozoon*. All other Kappa tests were conducted using WinPepi (Version 3.15 [29]) with a value of above 0.75 indicating excellent agreement, 0.60-0.74 good agreement, 0.40-0.59 indicating fair agreement and below 0.40 indicating poor agreement [30].

## Results

### *Trypanozoon*

A comparison of the sensitivity of the different PCR reactions in the diagnosis of *Trypanozoon*, species-specific PCR using TBR detected 63 (10.5% prevalence, 95% confidence interval (CI) = 8.2%-13.2%) parasitic events, GPI-PLC-PCR detected 46 (7.7% prevalence, 95% CI = 5.7%-10.1%) parasitic events while ITS-PCR detected 1 (0.2% prevalence, 95% CI = 0%-0.9%) parasitic events (see Table 2). TBR-PCR and PLC-GPI-PCR agreed positively on 46 samples with TBR-PCR detecting an additional 17 samples as being positive. TBR-PCR, GPI-PLC-PCR and ITS-PCR agreed positively on one sample with TBR-PCR and GPI-PLC-PCR identifying an additional 62 and 45 samples respectively. Fleiss’s Kappa from rating scores showed poor agreement between the three PCR reactions (Kappa = 0.3997, 95% confidence interval = 0.3535 to 0.4459) Kappa testing showed excellent agreement between TBR-PCR and GPI-PLC-PCR (Kappa = 0.83, 95% confidence interval = 0.75 to 0.91). There was poor agreement between ITS-PCR and both TBR-PCR (Kappa = 0.03, 95% confidence interval = −0.03 to 0.08) and GPI-PLC-PCR (Kappa = 0.04, 95% confidence interval = −0.04 to 0.11).

**Table 2:** **Comparison of species-specific and the modified ITS-PCR of Cox *et al *.**[20]**targeting African trypanosomes**

**PCR reaction**		**ITS-PCR**		**GPI-PLC**	
		**Positive**	**Negative**	**Positive**	**Negative**
TBR	Positive	1	62	46	17
	Negative	0	537	0	537
GPI-PLC	Positive	1	45		
	Negative	0	554		
TCS/TCF/TCK	Positive	0	0		
	Negative	13	587		
TV	Positive	8	26		
	Negative	9	557		

PCR reactions targeted the following genetic material TBR and GPI-PLC: *Trypanozoon* DNA; TCS, TCF, TCK: *Trypanosoma congolense* DNA; TV: *Trypanosoma vivax* DNA).

### *T. congolense*

Comparing the sensitivity (see Table 2) of the different PCR reactions in the diagnosis of *T. congolense*, species-specific PCR using TCS, TCF and TCK detected 0 (0% prevalence, 95% CI = 0%-0.6%) parasitic events while ITS-PCR detected 13 (2.2% prevalence, 95% CI = 1.2%-3.7%). The species-specific reactions (TCS-PCR, TCF-PCR and TCK-PCR) and ITS-PCR did not agree positively on any samples with ITS-PCR identifying 13 positives in total. Kappa testing showed poor agreement between the species-specific reactions (TCS-PCR, TCF-PCR and TCK-PCR) and ITS-PCR (Kappa = 0, 95% confidence interval = −0.00 to 0.00).

### *T. vivax*

Comparing the sensitivity (see Table 2) of the different PCR reactions in the diagnosis of *T. vivax*, specific PCR using TV detected 34 (5.7% prevalence, 95% CI = 4%-7.8%) parasitic events while ITS-PCR detected 17 (2.8% prevalence 95% CI = 1.5%-4.8%) parasitic events. TV-PCR and ITS-PCR only agreed positively on eight animals with TV-PCR and ITS-PCR identifying a further 26 and nine positives respectively. Kappa testing showed poor agreement between TV-PCR and ITS-PCR (Kappa = 0.29, 95% confidence interval = 0.12 to 0.45).

### Other trypanosome species

In addition to these pathogenic trypanosome species, ITS-PCR was able to identify non-pathogenic *T. theileri* species. The reaction diagnosed 46 animals (7.6%) infected with *T. theileri*. A species-specific PCR reaction that targets *T. theileri* satellite sequence of 500 bp size was developed by Rodrigues *et al*. [31] and was used in the current study but failed to amplify *T. theileri* DNA (data not shown).

## Discussion

The use of ITS-PCR in diagnosis of African trypanosomes allows the identification of several trypanosome species in the same reaction. This is both a saving in time and cost, as less PCR reactions need to be carried out to gain an understanding of the prevalence of trypanosomes in an area. Although FTA cards have simplified the collection and transport of blood samples [3], often DNA is not spread homogenously over the matrix [23]. Although this can be overcome by taking several punches from each FTA card this can be time consuming and expensive. Elution of DNA from the FTA card can improve the sensitivity of species-specific PCR reactions targeting trypanosomes [24]. In the current work we compared an ITS-PCR first described by Cox *et al*. [20] modified through the elution of DNA from the FTA card to species-specific PCR reactions. There was very little agreement for the three major African trypanosome species between these reactions. Although the modified ITS reaction did not perform well for *Trypanozoon* it performed much better for the detection of *T. congolense* when compared to species-specific reactions*.*

In this study, ITS-PCR identified only one (0.2%) sample to contain *Trypanozoon* while the species-specific reaction diagnosed 63 parasitic events. The difference between the sensitivity of ITS-PCR and species-specific PCR reactions observed here might relate to the frequency of the target within the parasite genome. The copy number of ITS target in the genome is 100–200 compared to 10,000 for TBR and the higher sensitivity of the latter reaction could be due to the increased copy number of the target DNA. However, good agreement was seen between TBR and GPI-PLC; with the latter targeting a single copy gene [12] it would seem doubtful that copy number alone is the reason for the lack of agreement between TBR and ITS PCR. Cox *et al*. [20] compared ITS-PCR with the same primers used in the current study and found that ITS-PCR identified 22% (n = 245) of samples positive for *Trypanozoon* DNA while TBR-PCR identified 21% (n = 245) of samples; these samples were collected on FTA®cards from cattle in Uganda but PCR was conducted directly on the discs after washing. However, more research by de Clare Bronsvoort *et al*. [22] had shown the species-specific TBR PCR reaction to be more sensitive than the Cox ITS-PCR when the PCR was seeded with washed discs. The differences between the sensitivities of the two reactions in the current study may be due to the fact that the ITS reaction was optimized to work on washed discs rather than eluted DNA. Although Cox *et al*. [23] showed a higher sensitivity when several punches were screened individually from the same card this is time consuming and costly. The advantage of using eluted DNA being that a greater volume of the FTA card can be sampled from a single PCR reaction [24]. In contrast, Njiru *et al*. [19] reported that ITS-PCR using other primer sets were comparable to the species-specific reactions where ITS primers detected 84.9% (373 cattle and 185 camels were tested) of the samples positive using species-specific primers. The higher sensitivity of ITS primers compared with species-specific primers could be attributed to the sample processing method, because Njiru *et al*. [19] extracted DNA from blood samples with Qiagen extraction kits and 2 μl of the extract was used as a template in the PCR reaction. Extraction of DNA directly from blood samples increased the sensitivity for the ITS reaction while elution of DNA from the FTA cards inhibits the ITS PCR. However, although Thumbi *et al*. [21] extracted of DNA from frozen blood samples using the salt-out procedure of Sandbrook *et al*. [32], the specific PCR and the two ITS reactions only detected low levels of infection with *Trypanozoon* (1.9-3.9%).

It was difficult to differentiate between *T. congolense* savannah type, *T. congolense* forest type and *T. congolense* kilifi type using ITS PCR even after prolonged separation time of the bands by electrophoresis. This was attributed to the band sizes of the amplified products of the three types are close to each other (1513 bp, 1422 bp and 1413 bp for *T. congolense* forest, *T. congolense* kilifi and *T. congolense* savannah, respectively). For this reason, any product with a band size above 1400 bp was classified as *T. congolense* species. The PCR reactions specific for *T. congolense* savannah, *T. congolense* forest and *T. congolense* kilifi were negative for all the examined samples with Kappa testing suggesting poor agreement between the two diagnostic PCR tests. Previously Cox *et al*. [20] had compared ITS-PCR with species-specific reactions on cattle blood samples collected on FTA®cards from Uganda. ITS-PCR identified six samples as *T. congolense* while only one sample was diagnosed using specific reactions for *T. congolense* savannah. The inability of the species-specific PCR reactions to identify the *T. congolense* species diagnosed with ITS-PCR could be explained by the existence of a new *T. congolense* sub-species which is similar to *T. congolense* savannah and *T. congolense* forest in ITS target, while there is no specific reaction to identify these isolates at present. Isoenzyme analysis by Gashumba *et al*. [33] had previously shown that *T. congolense* from Uganda was separated from other *T. congolense* isolates suggesting that further analysis of *T. congolense* from Uganda may be required.

In the present study, universal primers targeting the gene encoding *T. vivax* specific antigen were used for the diagnosis of *T. vivax* species. The universal primer set diagnosed 34 *T. vivax* infections while ITS-PCR identified 17 positive samples. Another set of primers targeting satellite DNA sequence were used and none of the samples were positive with these primers (data not shown). This was not unexpected since the primers targeting the satellite sequence are not present in some isolates of *T. vivax*[16]. Cox *et al*. [20] identified nine *T. vivax* infections in cattle blood samples using the primer set amplifying the satellite sequence, while ITS-PCR identified six *T. vivax* infections. Although Cox *et al*. [20] found little difference between the ITS-PCR and species-specific PCR, there were very few positive samples making comparisons difficult. It has recently been suggested that the species-specific primer sets for *T. vivax* may not accurately assess the level of infection within wild animal samples from Tanzania [34]. This agrees with the current work, where ITS PCR identified nine samples that were not identified by the species-specific primer set.

*T. theileri* was identified in 7.6% of samples using the modified nested ITS PCR. The prevalence obtained was similar to that obtained by Cox *et al*. [20] in a low *Trypanozoon* prevalence village (3%) but lower than that seen in a high *Trypanozoon* prevalence village (47%). As there were no positive results using the species-specific PCR reaction targeting *T. theileri* it was difficult to gauge the sensitivity of the modified ITS PCR in the current work.

## Conclusions

The present study suggests that for estimating prevalence of *Trypanozoon* within cattle populations, on samples that are collected on to FTA cards (that includes the zoonotic *T. b. rhodesiense*) species-specific primers are more suitable in terms of sensitivity. However, as species-specific primers did not identify any *T. congolense* and there were differences in the amplification of *T. vivax*, the ITS reactions of Cox *et al*. [20] and other ones that are available (for example Njiru *et al*. [19]) might be of great value for detecting these parasites.

## Competing interests

The authors declare they have no competing interests and the sponsors had no role in the study design, data collection and analysis, decision to publish, or preparation of the manuscript.

## Authors’ contributions

HAA carried out the study, the molecular approaches and participated in preparing the manuscript. KP and SCW designed the study supervised the molecular approaches and participated in preparing and editing the manuscript. ETM carried out the statistical analyses and participated in preparing and editing the manuscript. All authors read and approved the final version of the manuscript.
